# Exploring Oxidative Stress and Metabolic Dysregulation in Lung Tissues of Offspring Rats Exposed to Prenatal Polystyrene Microplastics: Effects of Melatonin Treatment

**DOI:** 10.3390/antiox13121459

**Published:** 2024-11-28

**Authors:** Hong-Ren Yu, Ching-Yi Tsai, Wei-Ling Chen, Po-Yu Liu, You-Lin Tain, Jiunn-Ming Sheen, Yi-Siang Huang, Mao-Meng Tiao, Chih-Yung Chiu

**Affiliations:** 1 Graduate Institute of Clinical Medicine, College of Medicine, Chang Gung University, Taoyuan 333, Taiwan; yuu2004taiwan@yahoo.com.tw (H.-R.Y.); weilingchen914@gmail.com (W.-L.C.); tainyl@hotmail.com (Y.-L.T.); ray.sheen@gmail.com (J.-M.S.); hiss2223@gmail.com (Y.-S.H.); 2 Department of Pediatrics, Kaohsiung Chang Gung Memorial Hospital, Kaohsiung 833, Taiwan; 3 Institute for Translational Research in Biomedicine, Chang Gung Memorial Hospital, Kaohsiung 833, Taiwan; cytsai@cgmh.org.tw; 4 School of Medicine, College of Medicine, The Center of Excellence for Metabolic Associated Fatty Liver Disease, National Sun Yat-sen University, Kaohsiung 807, Taiwan; poliu@mail.nsysu.edu.tw; 5 Department of Biomedical Science and Environmental Biology, Kaohsiung Medical University, Kaohsiung 807, Taiwan; 6 Division of Pediatric Pulmonology, Department of Pediatrics, Chang Gung Memorial Hospital at Linkou, Chang Gung University, Taoyuan 333, Taiwan; 7 Clinical Metabolomics Core Laboratory, Chang Gung Memorial Hospital at Linkou, Taoyuan 333, Taiwan

**Keywords:** microplastics, offspring, lung, metabolome, oxidative stress, melatonin

## Abstract

Metabolomics research provides a clearer understanding of an organism’s metabolic state and enables a more accurate representation of its functional performance. This study aimed to investigate changes in the metabolome of lung tissues resulting from prenatal exposure to polystyrene microplastics (PS-MPs) and to understand the underlying mechanisms of lung damage in rat offspring. We conducted metabolomic analyses of lung tissue from seven-day-old rat pups exposed to prenatal PS-MPs. Our findings revealed that prenatal exposure to PS-MPs led to significantly increased oxidative stress in lung tissues, characterized by notable imbalances in nucleic acid metabolism and altered profiles of specific amino acids. Furthermore, we evaluated the therapeutic effects of melatonin treatment on lung function in 120-day-old offspring and found that melatonin treatment significantly improved lung function and histologic change in the affected offspring. This study provides valuable biological insights into the mechanisms underlying lung damage caused by prenatal PS-MPs exposure. Future studies should focus on validating the results of animal experiments in humans, exploring additional therapeutic mechanisms of melatonin, and developing suitable protocols for clinical use.

## 1. Introduction

Plastic pollution is a significant global problem that is increasing at an alarming rate. Global plastic production increased from approximately 2 million tons in 2000 to over 400 million tons in 2019 [1]. This reflects the expansion of plastic consumption across various sectors, including packaging, construction, and electronics. An increase in plastic production is accompanied by an increase in the amount of plastic waste released into the environment. Microplastics (MPs) refer to plastic particles smaller than 5 mm, mainly produced through the breakdown of larger plastic items as a result of natural processes. Microplastics significantly affect various organisms, particularly in marine ecosystems. They can interfere with the growth of phytoplankton and reduce their photosynthetic capacity, which diminishes the primary productivity of ecosystems [2]. Fish and other aquatic species that consume microplastics may experience gastrointestinal and organ damage, which can lead to altered metabolism and behavior, further disrupting aquatic food chains [3]. These detrimental effects not only threaten the health of marine organisms, but may also disrupt the stability of entire ecosystems, thus ultimately posing risks to human health.

Human exposure to MPs occurs primarily through ingestion, inhalation, and skin contact [4]. Exposure to MPs leads to their accumulation in multiple tissues and, through several possible mechanisms, causes tissue damage, metabolic disorders, functional impairments, and disease exacerbation [4]. When focusing on the lungs, animal studies have shown that the inhalation of MPs disrupts the respiratory epithelial barrier, exacerbating inflammatory responses and oxidative stress in the lungs [5,6,7,8,9]. Several studies have shown that the inhalation of MPs may cause pulmonary fibrosis. In their study, Li et al. confirmed that the inhalation of tire wear MPs leads to restrictive ventilatory dysfunction, pulmonary inflammation, and lung fibrosis damage in C57BL/6 mice through the reorganization of the epithelial cell cytoskeleton [10]. The inhalation of polystyrene microplastics (PS-MPs) can induce oxidative stress and activate the Wnt/β-catenin signaling pathway, further inducing pulmonary fibrosis in mice [11]. Another study demonstrated that the intranasal administration of PS-MPs promoted ferroptosis in alveolar epithelial cells via the cGAS/STING signaling pathway, subsequently leading to pulmonary fibrosis [12]. However, most current research on the harm caused by MPs focuses on direct exposure experiments in animals, while investigations into the transgenerational effects of MPs on the health of organisms are still in their early stages.

Owing to the rapid organ development in embryos and fetuses, exposure to external adverse factors during this period can significantly increase the risk of disease later in life [13]. An observational study indicated that MPs sized between 5 and 10 µm could be detected in human placentas [14]. Furthermore, animal studies have shown that when mothers ingest MPs during pregnancy, they negatively affect the central nervous system, liver function, gut health, reproductive capabilities, and metabolic homeostasis [15]. Although researchers have studied the mechanisms by which MPs harm living organisms, the specific ways in which they affect these organisms are still not fully understood.

Metabolomic research offers direct insights into the physiological and pathological conditions within organisms, closely reflecting actual biological functions. This approach aids in understanding how toxins interfere with normal metabolic processes and the mechanisms underlying damage. This information is beneficial for assessing health risks and formulating appropriate prevention and treatment strategies. Reports on lung injury in offspring due to prenatal microplastic exposure are scarce. We aimed to explore the impact of prenatal MPs exposure on lung injury in offspring and potential prevention and treatment mechanisms through metabolomic research.

Melatonin, a natural indoleamine found in all aerobic organisms, possesses properties that regulates the circadian rhythm and provides cellular protection [13]. Melatonin has been shown to exhibit multiple pharmacological effects, including sleep regulation, antioxidant and anti-inflammatory properties, and endocrine regulation [14,15]. The pineal gland is the main source of melatonin in the human circulatory system [16]. Melatonin has been reported to potentially play a specific role in preventing and treating metabolic syndrome, cardiovascular diseases, and Alzheimer’s disease [17,18]. In our previous study, we found prenatal PS-MPs exposure to induce hepatic steatosis in offspring [19]. In another study, we confirmed that prenatal melatonin treatment could alleviate hepatic steatosis caused by prenatal dexamethasone exposure [20]. Therefore, in this study, we evaluated the efficacy of melatonin in treating damage to offspring lung tissue associated with prenatal and postnatal PS-MPs exposure.

## 2. Materials and Methods

### 2.1. Animals and Experimental Design

As previously reported, 16-week-old virgin female Sprague Dawley rats from BioLASCO were maintained in a controlled environment [21].

For the metabolomics study, dams were divided into two groups upon confirmation of pregnancy after mating: (1) In the control group: dams received PS-MPs-free drinking water. (2) MPs group: dams were administered drinking water containing PS-MPs (Dragon-green fluorescent carboxylated and non-functionalized PS-MPs; Bangs Laboratories; Fishers, IN, USA) (5 μm in size) at a concentration of 1000 μg per 1 L. The green fluorescent PS-MPs were uniformly distributed and consistently dispersed in an aqueous solution. The maximum excitation and emission wavelengths were 488 and 518 nm, respectively.

For the oxidative stress validation study, dams were divided into three groups: (1) the control group; (2) the MPL group, where dams were administered PS-MPs (100 g/L) in drinking water; and (3) the MPH group, where dams were administered drinking water containing PS-MPs (1000 μg/L). The dose-dependent effects of PS-MPs were determined.

The daily shaking of the drinking water bottles ensured that the rats were exposed to the PS-MPs in their drinking water. Male offspring were sacrificed on postnatal day 7 using Zoletil and xylazine, followed by saline perfusion.

For the melatonin treatment study, dams were divided into three separate groups: (1) control group; (2) MPs group: dams were administered drinking water containing PS-MPs (1000 μg/L) and male offspring also received drinking water containing PS-MPs (1000 μg/L) from gestational day 0 to postnatal day 120; (3) MPM group: dams were administered drinking water containing PS-MPs (1000 μg/L) and melatonin (40 mg/L) and male offspring also received drinking water containing PS-MPs (1000 μg/L) and melatonin (40 mg/L) from gestational day 0 to postnatal day 120. The male offspring were sacrificed on postnatal day 120 after the lung function test. Melatonin was prepared as shown in a previous report [22]. The average daily intake of melatonin was estimated to be 1 mg/kg/day.

A simple flowchart illustrating the study steps is shown in Supplementary Figure S1. The experimental protocol was approved by the Institutional Animal Care and Use Committee of the Chang Gung Memorial Hospital (approval number: 2019053001).

### 2.2. Histopathological Analysis

Lung tissues were preserved in 4% paraformaldehyde at 4 °C overnight, followed by dehydration through a series of ethanol gradients. Samples were cleared with xylene and embedded in paraffin wax. Sections from formalin-fixed tissues were sliced and stained using a hematoxylin and eosin (H&E) kit (ScyTek Laboratories, West Logan, WV, USA). Histological lesions were examined using a Leica DMI-3000 microscope equipped with a digital camera (Leica Biosystems, Buffalo Grove, IL, USA).

### 2.3. Immunohistochemistry

As previously reported [21], formalin-fixed tissue sections, each 4 μm thick, were affixed to polylysine-coated slides. Sections were deparaffinized using xylene, followed by rehydration in a series of alcohol and water baths. After staining with an anti-8-hydroxy-2-deoxyguanosine (8-OHdG) antibody (Santa Cruz Biotechnology, Inc., Santa Cruz, CA, USA) or anti-cleaved caspase-3 antibody (Cell Signaling Technology, Inc. Danvers, MA, USA) for 60 min at room temperature and a secondary antibody for 30 min after rinsing. Avidin and horseradish peroxidase H conjugated with 3,3′-Diaminobenzidine (DAB) were employed in order to enhance the visualization of the staining (Thermo Scientific Inc., TL-060-QHD; Waltham, MA, USA). The slides were examined using a Leica DMI-3000 microscope equipped with a digital camera. Staining was quantified using the ImageJ software (Fiji version 1.8.0) and evaluated using the Ultravision Quanto Detection System HRP DAB kit from Thermo Scientific Inc. (TL-060-QHD; Waltham, MA, USA) [21].

### 2.4. Sample Preparation and Non-Targeted LC-MS Metabolomics

LC-MS was used for metabolomics analysis [23]. In brief, 50 μL of tissue lysates was combined with 200 μL of pre-chilled methanol to precipitate any proteins present. Following this, the mixture underwent centrifugation at 12,000× *g* for 15 min, after which the supernatant was collected and dried under a nitrogen stream. The resulting residue was then diluted in 200 μL of 50% acetonitrile for subsequent LC-MS analysis. The liquid chromatographic separation was carried out using an ACQUITY UPLC BEH Amide column (1.7 μm, 2.1 × 150 mm; Waters, Milford, MA, USA) within an ACQUITY™ Ultra Performance Liquid Chromatography (UPLC) system (Waters Corp.). The column temperature was set to 45 °C, with a flow rate of 0.4 mL/min. The mobile phase comprised 0.1% formic acid in water (designated as phase A) and acetonitrile containing 0.1% formic acid (designated as phase B). Mass spectrometric analysis was performed using a Waters Q TOF-MS (SYNAPT G2S; Waters MS Technologies, Manchester, UK), operating in both positive and negative electrospray ionization (ESI) modes. The mass scan range was established from 50 to 1000 *m*/*z*. The desolvation gas was maintained at a flow rate of 800 L/h and heated to 500 °C. The source cone voltage was adjusted to 25 V. For the positive ion mode, the capillary voltage was set to 2.5 kV, while in negative mode, it was adjusted to 2 kV. Leucine encephalin served as the lock mass, with *m*/*z* values of 120.0813 and 556.2771 for positive mode, and 236.1035 and 554.2615 for negative mode. The identification of metabolites was carried out utilizing the Human Metabolome Database (HMDB) with high confidence. An orthogonal projection to latent structures discriminant analysis (OPLS-DA) model was employed for the analysis of metabolites. Significant metabolites were selected based on a variable importance in projection (VIP) value greater than 1.0. The analysis was conducted using SIMCA software (V16.0.2, Sartorius Stedim Data Analytics AB, Umea, Sweden). A *p*-value of less than 0.05 was considered indicative of significant differences between groups. Furthermore, the functional studies and analyses of these metabolites were conducted using the KEGG database to identify specific biological pathways.

### 2.5. Glutathione Determination

Total glutathione, oxidized glutathione (GSSG), and the reduced glutathione (GSH)/GSSG ratio in lung tissue were measured using kits obtained from Elabscience Biotechnology Inc., Houston, TX, USA (catalog #E-BC-K097-M). Briefly, after centrifuging the tissue extracts to remove debris, glutathione reductase was used to reduce GSSG to GSH. The GSH then reacted with 5,5′-dithiobis (2-nitrobenzoic acid) (DTNB) to produce GSSG and yellow 2-nitro-5-thiobenzoic acid (TNB). The total glutathione concentration (GSSG + GSH) was determined by measuring the optical density (OD) at 412 nm. To specifically determine the concentration of GSSG, GSH was removed from the sample using the scavenger reagent provided in the kit, and the same reaction principle was then applied to measure the remaining GSSG.

### 2.6. Western Blot

In accordance with the methodologies described in our previous report [21], lung tissue samples were prepared and subjected to Western blot analysis to assess protein expression levels. A total of 40 μg of protein was separated on a 12% polyacrylamide gel and then transferred to polyvinylidene fluoride membranes using a semidry transfer system. Then, the membranes were incubated with 5% skim milk (BD Biosciences, Franklin, NJ, USA) in TBST for 1 h at room temperature for blocking, followed by overnight incubation with anti-Malondialdehyde (MDA) antibody (ab27642, Abcam, Cambridge, MA, USA) at a dilution factor of 1:2000 at 4 °C. After washing, the membranes were treated with horseradish peroxidase (HRP)-conjugated GAPDH (ab181602, Abcam; 1:5000), and the signal was captured with the Bio-Rad Molecular Imager ChemiDocMP and analyzed using Image Lab version 5.0 software (Bio-Rad, Hercules, CA, USA).

### 2.7. Lung Function Test

Unanesthetized rats were placed in a plethysmograph (EMKA Technologies, Paris, France) for acclimatization. Baseline data on various parameterssuch as breathing frequency, tidal volume, inspiratory and expiratory durations, and airway resistance were recorded over a 3 min timeframe. Pulmonary function metrics were measured and averaged using a Biopac MP36 system (Biopac Systems Inc., Camino Goleta, CA, USA). To assess the consistency of the physiological data obtained from the plethysmograph, the measurements were repeated three times on the same animal using an unrestrained single-chamber device.

### 2.8. Statistical Analysis

Statistical analyses were performed using the Mann–Whitney U-test or *t*-test, as indicated. Values were expressed as mean ± standard error of the mean, and *p* < 0.05 was considered statistically significant. All statistical analyses were performed using SPSS 22.0 for Windows XP (SPSS, Inc., Chicago, IL, USA).

## 3. Results

### 3.1. The Consumtion of PS-MPs Pregnant Dams Can Impact the Lung Development of Their Offspring

**Figure 1:**
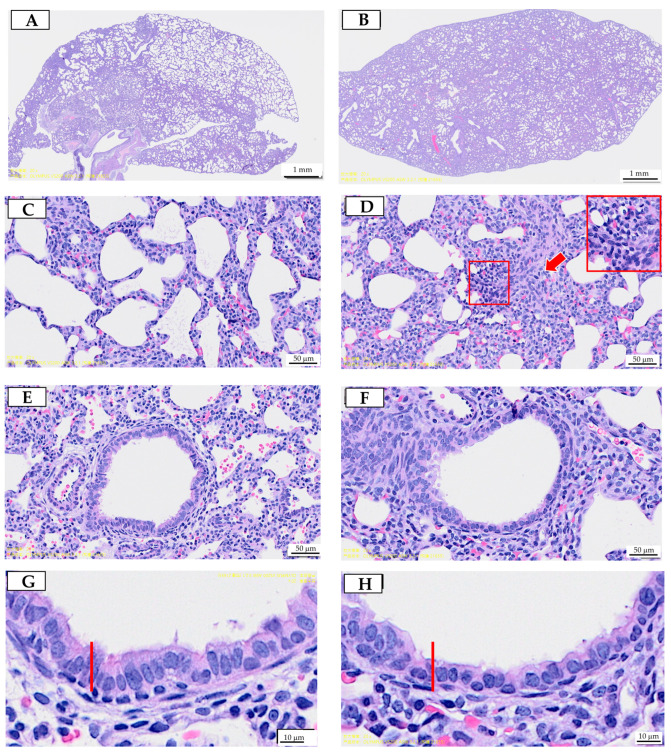
Prenatal polystyrene microplastics (PS-MPs) exposure leads to lung dysplasia in offspring during infancy. Histological manifestations of lung tissue from seven-day-old offspring with maternal microplastic particle exposure are shown. (**A**) Control group: under low-power microscopic field, the control group exhibited better pulmonary tissue aeration development. (**B**) PS-MPs exposure (1000 µg/L in drinking water for pregnant dams). (**C**) Zoomed-in images of Figure 1A. (**D**) Zoomed-in images of Figure 1B, showing the collapsed alveoli and thickened alveolar septa (red arrow). Magnification of boxed area detailing the inflammatory cells; cross-section of small airway from the (**E**) control group and (**F**) prenatal PS-MPs exposure group. (**G**) Zoomed-in images of (**E**). (**H**) Zoomed-in images of (**F**). The columnar epithelial cells in the airway of the prenatal PS-MPs exposure group are shorter and less developed, as indicated by the red marker representing the height of the control group cells. Each group comprised six animals. The yellow label at the bottom left of (**F**) and the top right of (**G**) is a specimen group annotation, with no other significance.

The H&E stain of lung tissue from the offspring is illustrated in Figure 1.

**Figure 2:**
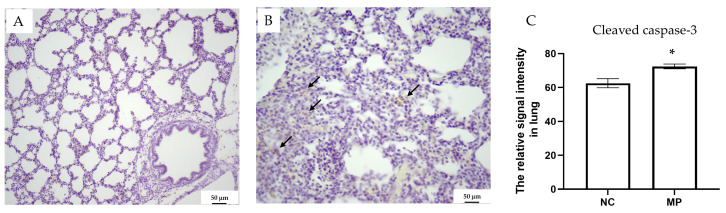
Cleaved caspase-3 staining for (**A**) the control and (**B**) PS-MPs group. The staining of cleaved caspase-3 is indicated with arrows. (**C**) Cleaved caspase-3 densities were quantified and compared between different groups. NC denotes the control group, while MP indicates PS-MPs exposure during pregnancy. Each group comprised six animals. Significant difference is indicated by *: *p* < 0.05.

Prenatal exposure to PS-MPs resulted in collapsed alveoli (Figure 1B), thickened alveolar septa (Figure 1D), and respiratory columnar epithelial cells with reduced height (Figure 1H) in offspring that were seven days old. The local accumulation and infiltration of inflammatory cells were also evident in lung tissue exposed to prenatal PS-MPs (Figure 1D). Cleaved caspase-3 expression has also been investigated to determine the role of apoptosis in offspring’s lung tissue with prenatal PS-MPs exposure. Under IHC staining, the lung tissue of seven-day-old offspring exposed to prenatal microplastic showed higher cleaved caspase-3 expression compared to the control group (Figure 2).

### 3.2. The Impact of Prenatal PS-MPs Exposure on the Metabolic Profile of Offspring Lung Tissue

#### 3.2.1. The Effects of Prenatal PS-MPs Exposure on the Lipid Metabolome of Offspring Lung Tissue

The lung tissues of the seven-day-old offspring with/without prenatal PS-MPs exposure were subjected to metabolite analysis using LC-MS to determine the altered metabolites that were affected by prenatal microplastic treatment. First, we investigated the changes in the lipid metabolome. A total of 3377 lipid-soluble metabolites were identified, with 25 entries meeting the false discovery rate (FDR) *p* < 0.05. Compared to the control group, 22 lipid-soluble metabolites were upregulated, whereas three lipid-soluble metabolites were downregulated in the group exposed to prenatal PS-MPs. PCA and hierarchical heat map clustering were performed based on lipid-soluble metabolites (Figure 2A,B), showing a clear separation of lipid-soluble metabolites between prenatal PS-MPs exposure group (MP) and the control group (NC).

After correction, 14 lipid-soluble metabolites showed statistically significant differences between the two groups (Table 1). Compared to the control group, among the identified differential lipid metabolites, only lysophosphatidylcholine (LPC) was significantly higher in the group exposed to prenatal PS-MPs, increased by 46,919 folds. The other 13 metabolites showed significant decreases compared to those in the control group. The least abundant was ceramide (Cer) 24:0;O2/14:1, which was 9.04 × 10^−6^ folds that in the control group.

**Figure 3:**
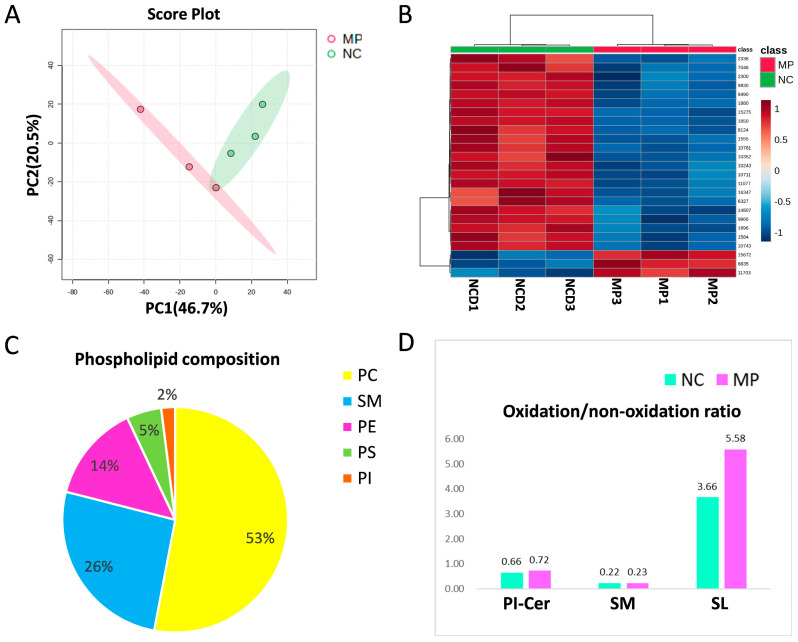
The differentially expressed lipid-soluble metabolites between the control group (NC) and prenatal PS-MPs exposure group (MP). (**A**) Principal component analysis (PCA) and (**B**) heatmap for lipid-soluble metabolites. The heatmap illustrates the top 25 metabolites with the most prominent differences between the two groups. In this display, the rows represent metabolite codes, columns represent samples, and the color intensity within each cell signifies the level of abundance (red = high; blue = low). (**C**) The composition of phospholipid from lung tissue of seven-day-old rat offspring. (**D**) Oxidized PI-Cer and SM in control group and in prenatal PS-MPs exposure group. Each group comprised three animals. Abbreviations: Cer, ceramide; PC, phosphatidylcholine; PE, phosphatidylethanolamine; PI, phosphatidylinositol; PS, phosphatidylserine; PA, phosphatidic acid; PG, phosphatidylglycerol; SM, sphingomyelin.

Phosphatidylcholine (PC) is the most abundant phospholipid in mammalian cells, accounting for 40–60% of the total phospholipids [24]. In the phospholipids analyzed in our samples, PC accounts for the highest proportion at 53%, followed by SM, PE, PS, and PI in descending order, with proportions of 26%, 14%, 5%, and 2%, respectively (Figure 3C). However, a 26% composition of SM is somewhat high compared to the general cell membrane profile. The elevated SM may suggest an adaptive or protective response to stabilize cell membranes under environmental stress. Within the phospholipids, only PI-Cer, SM and SL exhibit noticeable oxidative states. The prenatal PS-MPs exposure group showed higher oxidative states compared to the control group, along with a higher ratio of oxidized PI-Cer, SM, and SL than the control group (Figure 3D).

#### 3.2.2. Prenatal PS-MPs Exposure Alter Aqueous Metabolites of Offspring Lung Tissue

**Figure 4:**
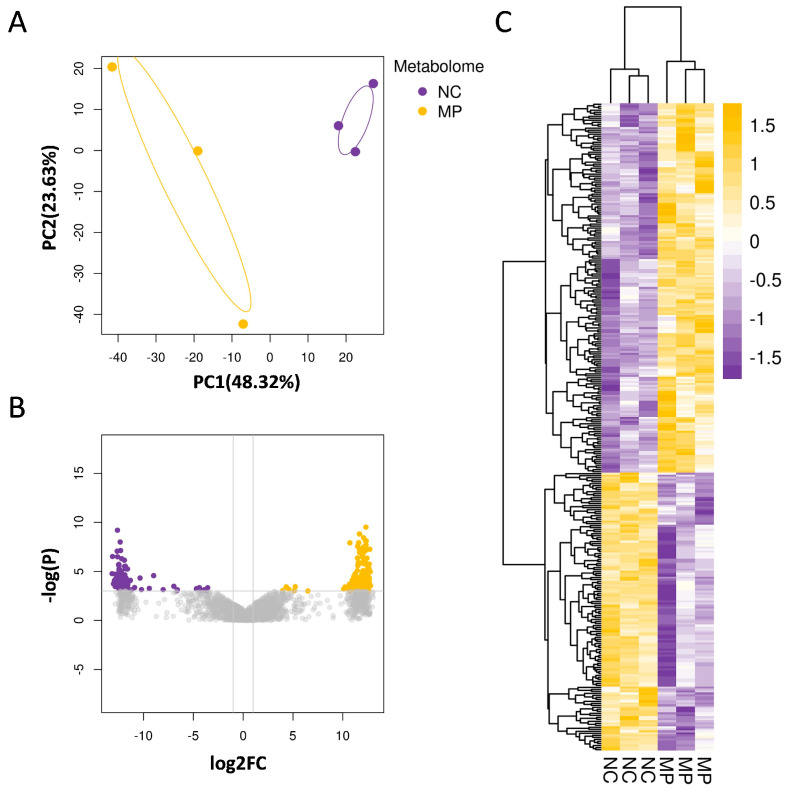
Prenatal exposure to PS-MPs altered the aqueous metabolite composition of offspring lung tissue. (**A**) The PCA displays the expression of aqueous metabolites in the control group (NC) and the prenatal polystyrene microplastics (PS-MPs) exposure group (MP). The yellow and purple dots represent three samples from the control group and the PS-MPs group, respectively. (**B**) The volcano plot demonstrates the differential expression of aqueous metabolites between the MPs and control groups. In this study, differential expression analysis of aqueous metabolites was performed, and a threshold of *p* < 0.05 and |fold change| ≥ 2 was used to identify significant differential metabolites. In the plot, the horizontal axis represents the fold change, while the vertical axis represents the adjusted *p*-value, with smaller values indicating more significant differences. Higher −log10 values correspond to higher statistical significance. (**C**) The heatmap shows the differential aqueous metabolites between the MPs group and control group. Increased aqueous metabolites were represented in yellow, and decreased aqueous metabolites were represented in purple, compared to the control group. Each group comprised three animals.

Next, lung tissues from seven-day-old offspring with and without prenatal PS-MPs exposure were subjected to aqueous metabolite analysis to determine the expression of aqueous metabolites affected by prenatal microplastic treatment. Similarly to the lipid phase metabolites, the aqueous metabolite profiles of the control and prenatal PS-NPs exposure groups displayed distinct separation patterns. The PCA plot and heatmap revealed differences in aqueous metabolites between the control and prenatal PS-MPs groups (Figure 3A,B). In this study, aqueous metabolites that met the threshold (*p* < 0.05 and |fold change| ≥ 2) were considered significant differential metabolites and used to assess the differences between the prenatal PS-MPs exposure group and the control group. Among the 2128 detected differential metabolites, 324 met the significance threshold of *p* < 0.05 and |fold change| ≥ 2. In comparison to the control group, there were 139 upregulated and 185 downregulated metabolites in the lung tissue of the seven-day-old offspring exposed to prenatal PS-MPs (Figure 4C).

Table 2 lists the top 20 statistically significant aqueous metabolites that were altered after prenatal microplastic exposure. Prenatal exposure to PS-MPs increased the levels of cytosine, hypoxanthine, creatinine, 5-oxoproline, and glutamine in lung tissues. Conversely, it decreased the levels of 2′,3′-dideoxyinosine, adenosine 5′-monophosphate, and 3-methylcytidine.

KEGG enrichment analysis was performed in order to identify the pathways associated with differential metabolite changes. Offspring exposed to PS-MPs exhibited significant effects on metabolic pathways in the lung tissue, particularly nucleotide (purine and pyrimidine), amino acid (alanine, aspartate, and glutamate), riboflavin, and nitrogen metabolism (Table 3). The altered metabolism of nucleotides (purines and pyrimidines) may weaken cellular proliferation and repair functions, thereby reducing the repair capacity of the lung tissue. Riboflavin, a component of various coenzymes, is crucial for energy release, and any impairment in energy metabolism in lung cells may result in decreased cellular function [25]. Additionally, abnormalities in amino acid metabolism, such as alanine, aspartate, and glutamate levels, may intensify inflammatory responses in the lungs. Furthermore, irregularities in nitrogen metabolism can lead to the excess production of reactive nitrogen species, triggering oxidative stress and inflammation. Overall, metabolic pathway abnormalities caused by prenatal exposure to PS-MPs may increase physiological stress, damage, and inflammatory responses in lung tissue, thereby negatively impacting lung function and overall health.

The increase in glutamine involved several important candidate pathways affected by prenatal PS-MPs exposure, including purine, pyrimidine, alanine/aspartate/glutamate, and glutathione metabolism. Given that glutathione plays a crucial role in maintaining redox homeostasis [26], and that we previously found oxidative stress involved in the liver damage of offspring caused by prenatal PS-MPs exposure, further research was conducted to investigate the role of oxidative stress in the lung damage of offspring resulting from prenatal PS-MPs exposure.

**Figure 5:**
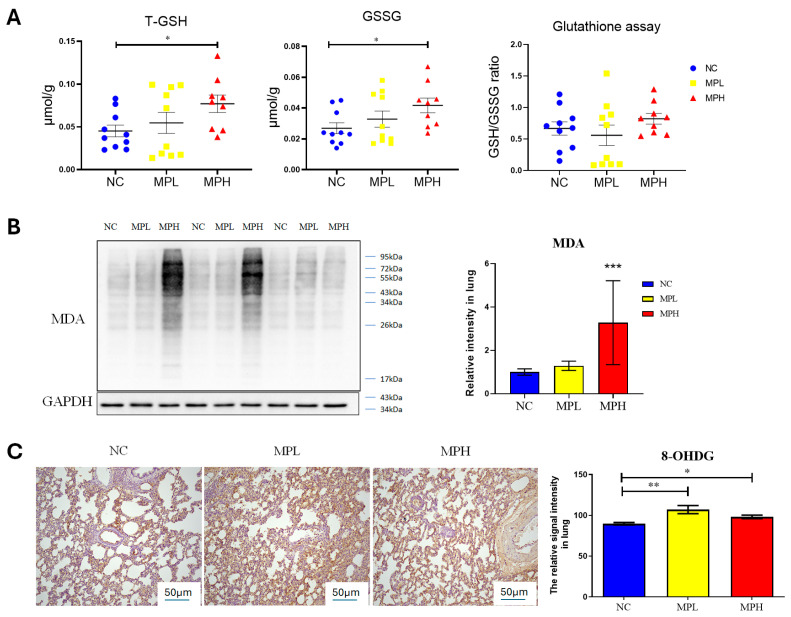
Oxidative stress in the lung tissue of pups. (**A**) Total glutathione (T-GSH), oxidized glutathione (GSSG), and ratio of reduced glutathione (GSH) to GSSG. Each group comprised 9–10 animals. (**B**) Western blot of Malondialdehyde (MDA) expression and semi-quantitative analysis. (**C**) 8-hydroxy-2-deoxyguanosine (8-OHdG) staining and semi-quantitative analysis. Magnification at 40× *: *p* < 0.05; **: *p* < 0.01; ***: *p* < 0.001. Each group comprised six animals.

To conduct the oxidative stress analysis, we added an additional group exposed to a lower dose of the prenatal PS-MPs to investigate dose-dependent effects. Initially, the total glutathione levels in the lung tissue of seven-day-old offspring were measured (Figure 4A). The group exposed to prenatal PS-MPs exhibited significantly higher levels of total glutathione and oxidized glutathione (GSSG) compared to the control group. However, the ratio of reduced glutathione (GSH) to GSSG was not significantly different. Compared to the control group, the lungs of offspring in the high-dose prenatal PS-MPs exposure group demonstrated a significant increase in MDA levels, which is a marker of lipid peroxidation and oxidative stress (Figure 4B). Additionally, in the analysis of 8OH-dG, a marker of DNA peroxidation and oxidative stress, both low- and high-dose prenatal PS-MPs exposure groups showed significantly elevated 8OH-dG expression in the lung tissues of the offspring (Figure 5C).

#### 3.2.3. Melatonin Treatment Partially Ameliorates Pulmonary Dysplasia and Lung Function in Adult Offspring Caused by Prenatal Plus Postnatal Exposure to PS-MPs

The next experiment was conducted in order to examine the lung damage caused by prenatal and postnatal PS-MPs exposure in rats and to identify whether melatonin intake can reduce the lung damage associated with such exposures. With prenatal and postnatal exposure to PS-MPs, inspiratory time (Ti), expiratory time (Te), and specific airway resistance (sRaw) increased, whereas minute ventilation (MV) and breathing frequency (F) decreased (Table 4). Melatonin treatment improved the increased Ti and Te and enhanced the decreased peak expiratory flow (PEF), peak inspiratory flow (PIF), and breathing frequency (F).

**Figure 6:**
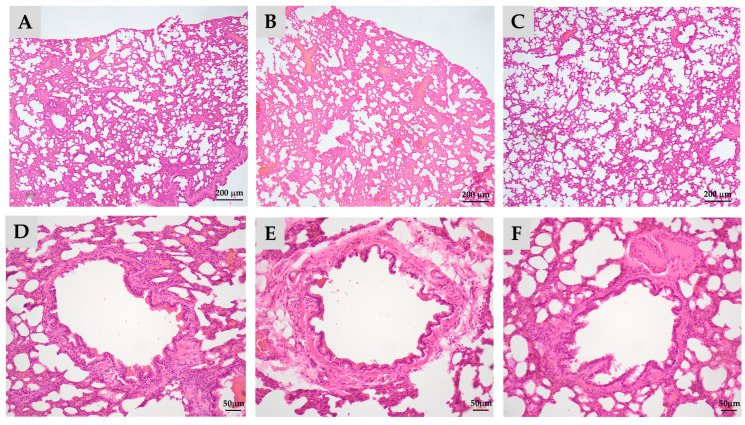
Melatonin treatment partially ameliorates pulmonary hypoplasia in adult offspring caused by prenatal and postnatal exposure to polystyrene microplastics (PS-MPs). Histological manifestations of lung tissue from 120-day-old offspring under low-power microscopic field. (**A**) Control group; (**B**) prenatal plus postnatal PS-MPs exposure group showing the alveolar collapse and hypertrophied alveolar septa; (**C**) prenatal plus postnatal PS-MPs exposure with melatonin treatment group; (**D**) zoomed-in images of (**A**); (**E**) zoomed-in images of (**B**) showing thickened connective tissue beneath the epithelial layer; (**F**) zoomed-in images of (**C**). Each group comprised three animals.

Following the assessment of lung function, we proceeded with histological analyses to further investigate the structural changes in lung tissue of 120-day-old offspring with prenatal plus postnatal PS-MPs exposure. We found that, similarly to the seven-day-old offspring exposed to prenatal PS-MPs, combined prenatal and postnatal exposure to PS-MPs led to alveoli collapse, thickened alveolar septa, and increased connective tissue below the epithelial layer in the lung tissue of the 120-day-old offspring (Figure 6).

## 4. Discussion

Retrospective analyses have suggested that prenatal PS-MPs exposure adversely affects the central nervous system, liver, intestines, reproductive system, and muscle tissue of the offspring [27].

In a previous study, we reported that after pregnant dams ingest PS-MPs, their offspring exhibit an increase in body weight and the shortening of the ileal villi [19]. Prenatal exposure to microplastics also leads to an increase in lipid accumulation in the livers of offspring compared to that in the control group [19]. In this study, we investigated the effects of prenatal PS-MPs exposure on the lung tissues of infant offspring. Our findings indicate that prenatal exposure to PS-MPs significantly affects the development of lung tissue in offspring. This study employed metabolomic techniques to analyze the potential mechanisms by which prenatal PS-MPs influence the development of lung tissue in the offspring. Compared to the control group, prenatal PS-MPs exposure notably affected the growth and biological processes in the lung tissue of the offspring. The pathways affected by prenatal PS-MPs were primarily enriched in purine and pyrimidine metabolism, specific amino acid metabolism, and nitrogen metabolism. Abnormalities in these processes, particularly in the metabolism of purines, pyrimidines, and amino acids (aspartate, glutamate, and threonine), can lead to oxidative stress [28,29,30], which supports our findings that maternal exposure to prenatal PS-MPs resulted in increased oxidative phospholipid levels in the lung tissue of the offspring.

Phospholipids and cholesterol are crucial components of double-layered membranes in mammalian cells. Phosphatidylcholine (PC) and phosphatidylethanolamine (PE) are major phospholipid species in mammalian cell membranes, whereas minor components include phosphatidylinositol (PI), phosphatidylserine (PS), phosphatidic acid (PA), phosphatidylglycerol (PG), and sphingomyelin (SM) [22]. Apart from their structural role in the membrane, phospholipids have also been shown to play essential roles in various cellular processes such as membrane transport, cell signaling, cell proliferation and differentiation, cell migration, and apoptosis. The observation that the group exposed to prenatal PS-MPs exhibited increased levels of certain oxidative phospholipid components compared with the control group suggests an elevation in oxidative stress within the cells.

Metabolic reprogramming has been detected in the lung tissues of patients with bronchopulmonary dysplasia, chronic obstructive pulmonary disease (COPD), and pulmonary fibrosis. In a cigarette smoke-induced COPD mouse model, FAM13A (family with sequence similarity 13 member A) was found to promote fatty acid oxidation by activating sirtuin 1 and increasing the expression of carnitine palmitoyl transferase 1A (CPT1A) [31]. Human studies further indicate that patients with severe COPD exhibit significantly elevated glycolysis and oxidation levels compared to control subjects [32]. Additionally, research highlights that alteration in polyamine metabolism, glycolysis, mitochondrial β-oxidation, and the tricarboxylic acid cycle could play crucial roles in the development of idiopathic pulmonary fibrosis [33]. However, the role of metabolic reprogramming and molecular mechanisms underlying these diseases are not yet fully understood. Purines (adenine and guanine) and pyrimidines (cytosine, thymine, and uracil) are fundamental components of nucleic acids that are involved in the replication of genetic material, gene transcription, protein synthesis, and energy transfer (for example, ATP) [34]. The interactions between purines and pyrimidines are crucial for the transmission of genetic information and cellular metabolism.

Glutamine elevation reflected the convergence of key metabolic pathways affected by prenatal PS-MPs exposure, including purine, pyrimidine, alanine/aspartate/glutamate, and glutathione metabolism. Glutamine is also involved in the synthesis of glutathione and serves as a carbon source for oxidative processes in certain cells [35,36]. In most cells, glutamine metabolism is facilitated by glutaminase, which converts glutamine to glutamate. Moreover, glutamine may play a direct role in cellular antioxidant defense mechanisms [37]. In response to stress, lung tissue utilizes glutamine synthetase to synthesize glutamine from glutamate and ammonia, thereby ensuring the stability of glutamine levels [38,39]. The release of stress-induced glutamine from the lungs is regulated by glucocorticoids and protein stability [38,39]. In a mouse model of endotoxin-induced acute respiratory distress syndrome, intravenous glutamine administration reduced lung inflammation and extracellular trap release, improved lung elastance, and mitigated alveolar collapse [40]. These results suggest that prenatal PS-MPs exposure leads to a compensatory increase in glutamine levels in offspring lung tissue in response to stress. This compensatory phenomenon is also reflected in the increase in the total glutathione protein of the γ-glutamyl cycle.

Glutathione, which is composed of glutamate, cysteine, and glycine, is vital for protecting cellular components from damage caused by reactive oxygen species and heavy metals. It is essential to prevent oxidative damage, mitigate the toxicity of xenobiotic electrophiles, and maintain the redox balance [26]. In healthy cells and tissues, reduced glutathione (GSH) is the predominant form, whereas oxidized glutathione (GSSG) is relatively less abundant. The GSH/GSSG ratio is an important indicator of cellular oxidative stress. When this ratio increases, the concentration of GSSG is elevated relative to GSH, which typically indicates that the cells are under a greater oxidative pressure [41]. When phospholipids containing polyunsaturated fatty acids are exposed to oxidative attack, oxidized phospholipids are formed [42]. In our study, we found that prenatal exposure to PS-MPs resulted in elevated oxidation of cell membrane phospholipids in the lung tissue of the offspring. These findings indicate that prenatal exposure to PS-MPs increases oxidative stress. The elevated oxidative stress in the lung tissue of offspring prenatally exposed to PS-MPs was further corroborated by increased levels of 8-OHdG and MDA.

**Figure 7:**
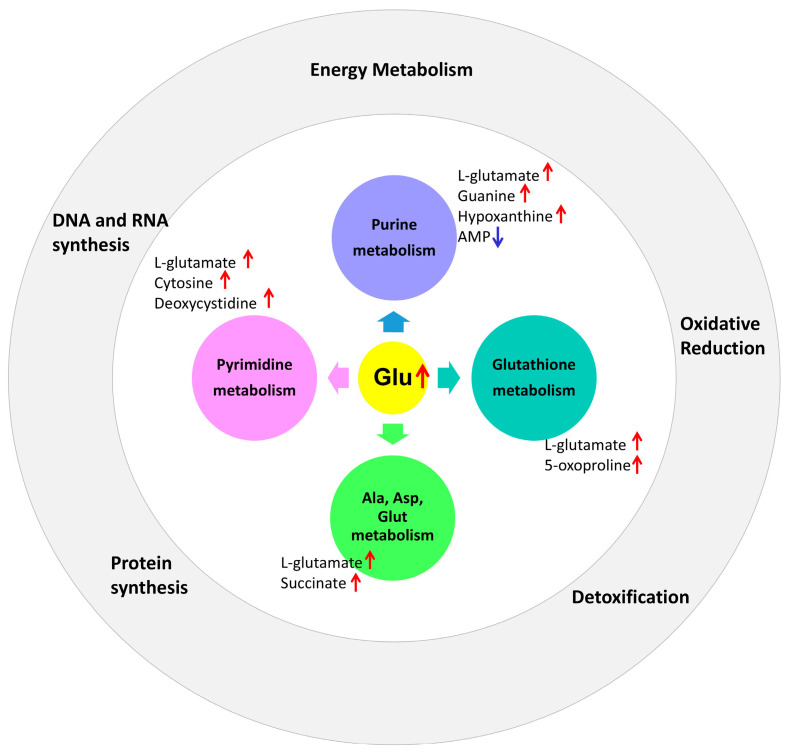
Prenatal microplastic exposure affects the metabolic pathways of purine, pyrimidine, alanine/aspartate/glutamate, and glutathione in the lung tissue of the offspring, along with their corresponding biological functions. The red upward arrow represents an increase, while the blue downward arrow represents a decrease.

In addition to an increase in glutamate, prenatal PS-MPs exposure influences the pyrimidine metabolic pathway, resulting in elevated cytosine and deoxycytidine levels. In the purine metabolic pathway, increased levels of guanine and hypoxanthine were observed, whereas AMP levels decreased. Furthermore, in the alanine/aspartate/glutamate metabolic pathway, prenatal microplastic exposure leads to elevated levels of glutamate and succinate in the lung tissues of offspring. In the glutathione metabolic pathway, increased 5-oxoproline levels were detected in the lung tissues of the offspring (Figure 7).

As an antioxidant, melatonin and its metabolites have a broad capability to neutralize superoxide anions (O_2_•^−^), hydroxyl radicals (•OH), hydrogen peroxide (H_2_O_2_), hypochlorous acid (HOCl), nitric oxide (NO), and peroxynitrite anions (ONOO^−^) [15]. Melatonin is an immunomodulatory agent with anti-inflammatory properties [18,43]. This effect may occur through the inhibition of nuclear factor kappa B (NF-κB) binding to DNA, thereby reducing the synthesis of pro-inflammatory cytokines by inhibiting cyclooxygenase [43]. Caspase-3 is a cysteine protease that plays an important role in the apoptosis and DNA damage response [44]. It generally exists in an inactive precursor form. When cells receive apoptotic signal, it is activated by other caspases to form cleaved caspase-3 form. The active form initiates cellular changes that ultimately lead to cell death and other downstream responses. Research demonstrates that melatonin protects various cell types by reducing caspase-3 activation, a critical executor of apoptosis. In a rat model of sepsis induced by cecal ligation and puncture, melatonin treatment significantly enhanced the survival rates and mitigated lung damage by decreasing caspase-3 expression [45]. In our study, we observed increased localized accumulation and infiltration of inflammatory cells in lung tissue exposed to prenatal PS-MPs. Given that melatonin has both antioxidant and anti-inflammatory effects, it is likely that melatonin improves lung function in offspring exposed to prenatal PS-MPs through both antioxidant and anti-inflammatory mechanisms.

Although our research identified metabolites related to prenatal exposure to PS-MPs in the lung tissues of neonates as well as their potential mechanisms and associations, certain limitations must be considered when interpreting these findings. Previous estimates have suggested that humans could ingest between 0.1 and 5 g of microplastics each week via various exposure routes [46]. In this dosage range, a rat weighing 200 g would therefore consume approximately 10 μg to 2 mg of microplastics daily. Considering that rats generally drink around 10 milliliters per 100 g of body weight per day, a concentration of 1000 micrograms per liter of microplastics in their drinking water results in a relatively low intake daily when compared to potential human exposure situations. However, human exposure to microplastics in natural environments involves diverse mixtures [27], making it more complex to design experiments aimed at investigating the effects of microplastics on human health. Second, in addition to its antioxidant properties, melatonin has other potential functions. Therefore, further research is necessary to reveal other specific mechanisms through which melatonin prevents and mitigates the damage caused by PS-MPs.

This study also has another limitation that may affect the overall interpretation and understanding of the relationship between prenatal microplastic exposure and offspring health outcomes. The lack of assessment of changes in the gastrointestinal histology of the dams means that it is not possible to rule out the absorption of PS-MPs due to damage to the intestinal barrier. However, the existing literature reports that the prolonged ingestion of PS-MPs in mice leads to a reduction in the height and surface area of intestinal villi, as well as a downregulation of related tight junction proteins, which may result in compromised intestinal barrier function [47]. Further research evaluating the intestinal histology of the dams could provide a more comprehensive understanding of the overall impact of prenatal microplastic exposure on both the mother and offspring. The current findings do not offer clear conclusions regarding whether the changes in the lung tissue induced by prenatal exposure to PS-MPs are irreversible or recoverable. This uncertainty arises from the study design, which did not include offspring prenatally exposed to PS-MPs during adulthood. In clinical applications, melatonin is typically administered at doses of 2–5 mg per day. However, studies based on animal research indicate that several potential therapeutic effects of melatonin may require higher doses in the range of 40–100 mg per day to manifest, which are rarely used in clinical settings [48]. Further human studies are required to investigate the effects of melatonin at higher doses.

## 5. Conclusions

Through metabolomic analysis, we expanded our understanding of the effects of prenatal PS-MPs exposure on toxicity in offspring lung tissues. Metabolomic analyses revealed that prenatal PS-MPs exposure caused significant oxidative stress and disrupted the nucleic acid metabolism and amino acid profiles in the lung tissues of seven-day-old rat pups. In addition, melatonin treatment significantly improved lung function in 120-day-old rats exposed to both prenatal and postnatal PS-MPs. These findings suggest that melatonin may be an effective therapeutic agent for mitigating lung damage caused by environmental pollutants, such as PS-MPs. Future studies should aim to validate these findings in humans, investigate the broader therapeutic mechanisms of melatonin, and establish effective clinical protocols to enhance its application in therapeutic settings.

## Figures and Tables

**Table 1:** The VIP score and fold change in lipid-soluble metabolites significantly differentially expressed between lung tissue of 7-day-old male spring rat with and without prenatal PS-MPs exposure.

Metabolites	VIP Score *	Fold Change ^†^	*p*
PC 35:4	1.19	1.37 × 10^−5^	0.021
PE 8:0_28:4	0.91	1.71 × 10^−5^	0.028
LPC 30:1	0.74	46,919	0.030
SM 32:3;O3	1.42	1.23 × 10^−5^	0.032
Cer 24:0;O2/14:1	2.09	9.04 × 10^−6^	0.041
PC 17:4_21:4	1.77	1.33 × 10^−5^	0.041
PC 10:0_26:6	1.25	1.07 × 10^−5^	0.041
TG 36:2_16:4_16:4	2.48	1.02 × 10^−5^	0.041
PC 42:11	1.70	1.44 × 10^−5^	0.041
PC O-16:1_24:6	1.11	8.22 × 10^−6^	0.041
SL 15:2;O/18:5	1.38	1.16 × 10^−5^	0.042
PC O-20:0_28:2	3.40	7.93 × 10^−6^	0.046
NAGlySer 24:6;O(FA 19:5)	1.40	1.15 × 10^−5^	0.048
TG 18:5_21:5_18:0;O4	1.62	1.00 × 10^−5^	0.048

* VIP scores were obtained from partial least squares discriminant analysis (PLS-DA). ^†^ Fold changes were calculated by dividing the value of metabolites in rats with prenatal PS-MPs exposure by control group. VIP, variable importance in projection; FDR, false discovery rate; PC, phosphatidylcholine; PE, phosphatidylethanolamine; LPC, lysophosphatidylcholine; SM, sphingomyelin; Cer, ceramide; TG, triglyceride; SL, saccharolipid; NAGly, N-acyl glycine; Ser, serine. Each group comprised three animals.

**Table 2:** The aqueous metabolites differentially expressed between lung tissue of seven-day-old male offspring rat with and without prenatal PS-MPs exposure.

	Aqueous Metabolites		
Metabolites	VIP Score *	Fold Change	*p*
Cytosine	1.76	5220.8	<0.001
2′,3′-Dideoxyinosine	1.92	0.000177	0.003
N-Methylisoleucine	1.35	4062.9	0.008
Hypoxanthine	1.23	3840.1	0.013
Adenosine 5′-monophosphate	2.23	0.000141	0.015
Raffinose	1.35	3543	0.016
Succinic acid	1.08	3228.9	0.016
Creatinine	1.41	4866.1	0.017
2′-Deoxycytidine	1.05	3884.5	0.026
3-Methylcytidine	1.5	0.000238	0.026
alpha-Galactosamine-1-phosphate	0.78	1881.3	0.027
Hydromorphone	1.12	3913.5	0.027
Pyroglutamic acid (5-oxoproline)	2.00	5696.8	0.027
Methohexital	0.74	2085.8	0.031
Guanine	1.64	4765.5	0.032
Phenylacetylglutamine	1.05	2939.6	0.032
Betaine	1.02	3131.1	0.034
Riboflavin	1.47	4449.9	0.035
Glutamine	1.91	5635.2	0.038
N8-Acetylspermidine	1.39	3217.7	0.041

* Variable importance in projection (VIP) scores were obtained from PLS-DA. Fold changes were calculated by dividing the value of metabolites in seven-day-old offspring rats with prenatal PS-MPs exposure by the value of those without prenatal PS-MPs exposure. Each group comprised three animals.

**Table 3:** Metabolic pathways and functional analysis of metabolites in offspring rats with prenatal polystyrene microplastics (PS-MPs) exposure.

Pathway Name	Metabolites	Total	Hits	*p*	Function
Purine metabolism	Glutamine, AMP, Hypoxanthine, Guanine	70	4	0.001	Nucleotide metabolism
Alanine, aspartate, and glutamate metabolism	Glutamine, Succinate	38	2	0.012	Amino acid metabolism
Pyrimidine metabolism	Glutamine, Deoxycytidine	39	2	0.023	Nucleotide metabolism
Riboflavin metabolism	Riboflavin	4	1	0.025	Metabolism of cofactors and vitamins
Nitrogen metabolism	Glutamine	6	1	0.037	Amino acid metabolism

“Total” is the total number of compounds in the pathway; “Hits” is the actual matched number from the user-uploaded data.

**Table 4:** Metabolic pathways and functional analysis of metabolites in offspring rats with prenatal polystyrene microplastics (PS-MPs) exposure.

	MV (mL)	*p* (msec)	F (bpm)	EIP (msec)	EEP (msec)	dT (msec)	SRaw (cm H_2_O.s)	SGaw (1/cm H_2_O.s)	EF50 (mL/s)
NC	595.33 ± 155.32	273.04 ± 33.22	227.46 ± 31.79	13.43 ± 1.25	12.71 ± 1.77	1.07 ± 0.76	1.03 ± 0.74	0.42 ± 0.19	25.94 ± 7.61
MP	480.81 ± 103.51 *	358.45 ± 31.82 **	173.73 ± 14.80 **	17.84 ± 1.78 **	18.56 ± 2.70 **	2.24 ± 0.32 **	2.18 ± 0.31 **	0.51 ± 0.07	19.52 ± 5.28
MPs + M	706.12 ± 229.52	302.95 ± 13.40 ^†^	202.21 ± 8.66 ^†^	13.23 ± 0.75 ^††^	14.42 ± 0.82 ^††^	2.00 ± 1.32	1.94 ± 1.29	0.47 ± 0.11	28.18 ± 10.81 ^†^
	Ti (msec)	Te (msec)	PIF (mL/s)	PEF (mL/s)	TV (mL)	EV (mL)	NTV (mL)	NEV (mL)	RT (msec)
NC	153.32 ± 22.58	128.73 ± 20.50	24.05 ± 6.32	29.10 ± 7.55	2.69 ± 0.85	2.72 ± 0.90	1.91 ± 0.32	1.91 ± 0.32	83.29 ± 12.19
MP	172.97 ± 11.58 *	186.15 ± 25.21 **	21.09 ± 3.36	21.95 ± 5.88	2.79 ± 0.56	2.82 ± 0.56	1.49 ± 0.30	1.49 ± 0.30	119.08 ± 16.53 **
MPs + M	155.87 ± 3.04 ^†^	147.99 ± 11.36 ^†^	28.76 ± 8.35 ^†^	34.09 ± 9.76 ^††^	3.51 ± 1.07	3.56 ± 1.07	1.88 ± 0.60	1.88 ± 0.60	94.59 ± 6.65 ^††^

Data are the mean ± SD of five experiments in triplicate. * NC (control) vs. MPs (PS-MPs exposure) group, * *p* < 0.05, ** *p* < 0.01; MPs group vs. MPs + M (PS-MPs exposure + melatonin treatment) group, ^†^ *p* < 0.05, ^††^ *p* < 0.01. Abbreviations: F, breathing frequency; TV, tidal volume; MV, minute ventilation; Ti, inspiratory time; Te, expiratory time; PIF, peak inspiratory flow; PEF, peak expiratory flow; EV, expiratory volume; NTV, nasal tidal volume; NEV, nasal expiratory volume, EIP, end inspiratory pause; EEP, end expiratory pause; dT, time delay; sRaw, specific airway resistance; sGaw, specific airway conductance; EF50, flow at mid-tidal expiratory volume. Each group comprised 9–10 animals. Data are the mean ± SD of triplicate.

## Data Availability

The datasets generated and/or analyzed during the current study are not publicly available due to institutional requirements but are available from the corresponding author upon reasonable request.

## Supplementary Materials

The following supporting information can be downloaded from https://www.mdpi.com/article/10.3390/antiox13121459/s1. Figure S1. Simple flowchart illustrating the study steps. (NC = control group; MPL = low-dose PS-MPs exposure (100 μg/L in drinking water); MPH = high-dose PS-MPs exposure (1000 μg/L in drinking water); MPM = high-dose PS-MPs exposure and melatonin treatment.
