# Supplementary figures and images for: Exploring Oxidative Stress and Metabolic Dysregulation in Lung Tissues of Offspring Rats Exposed to Prenatal Polystyrene Microplastics: Effects of Melatonin Treatment
Source: Antioxidants (Basel). 2024 Nov 28;13(12):1459. doi: 10.3390/antiox13121459 (PMC11672973; doi:10.3390/antiox13121459)

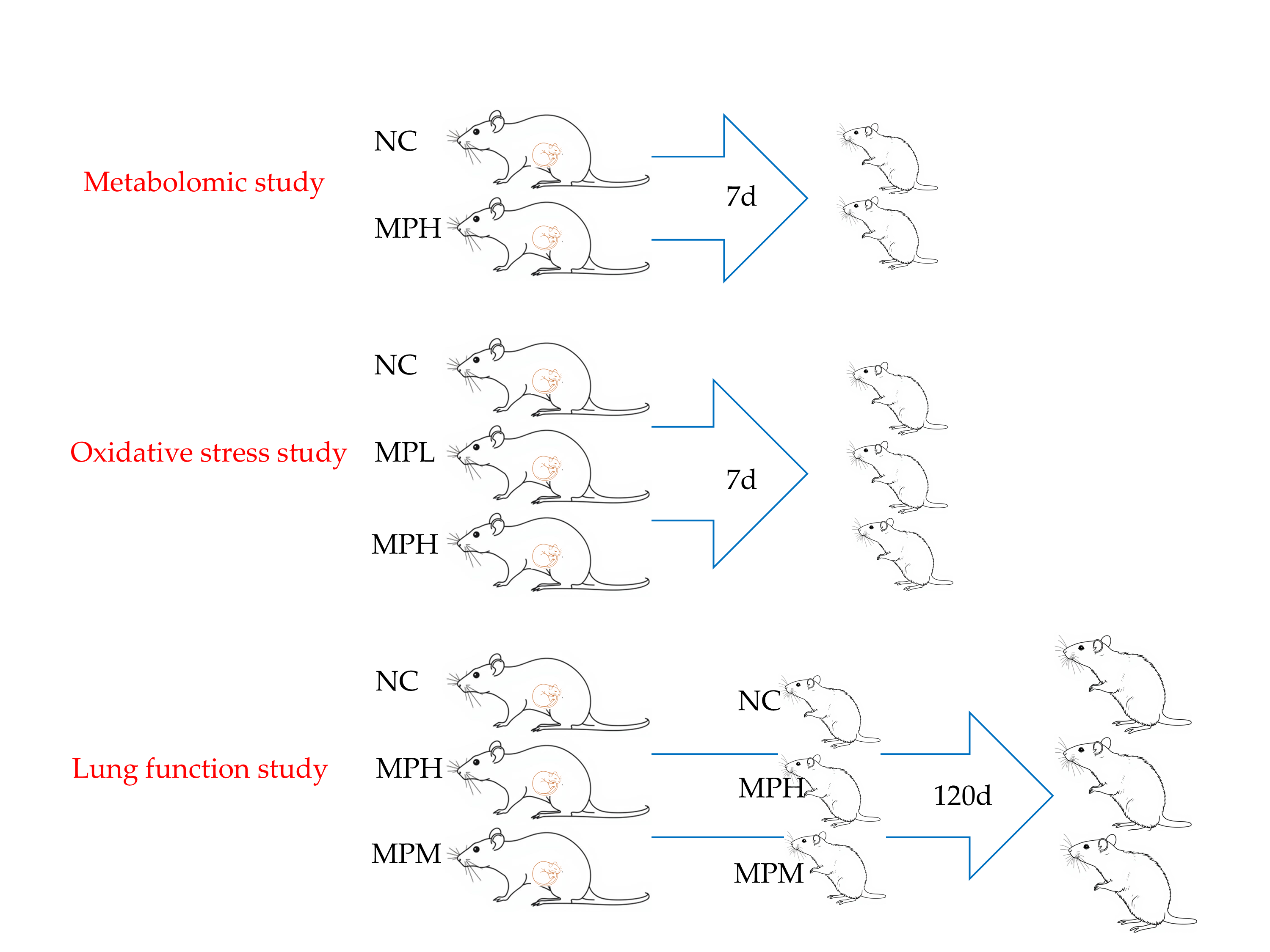

Supplement: Supplementary file 1 [file antioxidants-13-01459-s001.zip › antioxidants-3287945-supplementary.tif]
